# Lucid dreaming increased during the COVID-19 pandemic: An online survey

**DOI:** 10.1371/journal.pone.0273281

**Published:** 2022-09-14

**Authors:** Kelly P., Macêdo T., Felipe T., Maia M., Suely A., Herminia G., Jatahy M., Gomes L., Barroso L., Lima T. Z., Holzinger B., Ribeiro S., Mota-Rolim S.

**Affiliations:** 1 Brain Institute - Federal University of Rio Grande do Norte, Natal, Brazil; 2 Department of Psychology - Federal University of Rio Grande do Norte, Natal, Brazil; 3 Bioinformatics Multidisciplinary Environment (BioME) - Federal University of Rio Grande do Norte, Natal, Brazil; 4 Department of Engineering - Federal University of Rio Grande do Norte, Natal, Brazil; 5 Philosophy Department - Federal University of Pernambuco, Recife, Brazil; 6 Philosophy Department - Federal University of Paraná, Curitiba, Brazil; 7 Department of Psychopedagogy - Federal University of Paraiba, João Pessoa, Brazil; 8 Health Sciences Department - State University of Rio Grande do Norte, Mossoró, Brazil; 9 Institute for Consciousness and Dream Research, Vienna, Austria; 10 Physiology and Behavior Department - Federal University of Rio Grande do Norte, Natal, Brazil; 11 Onofre Lopes University Hospital - Federal University of Rio Grande do Norte, Natal, Brazil; University of Nairobi, KENYA

## Abstract

The COVID-19 pandemic changed people’s lives all over the world. While anxiety and stress decreased sleep quality for most people, an increase in total sleep time was also observed in certain cohorts. Dream recall frequency also increased, especially for nightmares. However, to date, there are no consistent reports focusing on pandemic-related changes in lucid dreaming, a state during which dreamers become conscious of being in a dream as it unfolds. Here we investigated lucid dreaming recall frequency and other sleep variables in 1,857 Brazilian subjects, using an online questionnaire. Firstly, we found that most participants (64.78%) maintained their lucid dream recall frequency during the pandemic, but a considerable fraction (22.62%) informed that lucid dreams became more frequent, whereas a smaller subset (12.60%) reported a decrease in these events during the pandemic. Secondly, the number of participants reporting lucid dreams at least once per week increased during the pandemic. Using a mixed logistic regression model, we confirmed that the pandemic significantly enhanced the recall frequency of lucid dreams (p = 0.002). Such increase in lucid dreaming during the pandemic was significantly associated with an enhancement in both dream and nightmare recall frequencies, as well as with sleep quality and symptoms of REM sleep behavior disorder. Pandemic-related increases in stress, anxiety, sleep fragmentation, and sleep extension, which enhance REM sleep awakening, may be associated with the increase in the occurrence of lucid dreams, dreams in general, and nightmares.

## 1 Introduction

On March 11, 2020, the World Health Organization officially declared COVID-19 a worldwide pandemic [1]. COVID-19 is a highly contagious disease caused by the coronavirus SARS-COV2. Its main symptoms are fever, cough, fatigue, and endotheliitis, which can lead to death due to pneumonia with microembolism [2–4]. The pandemic caused major psychological distress [5–7], with a negative impact on social life due to quarantines and lockdowns. The COVID-19 crisis is a global public health emergency without precedent in the past several decades [8], with overwhelming negative impacts ranging from agriculture [9] and gender equality [10] to consumption habits [11], poverty rates [12], education [13] and mental health [14].

In line with so many changes, sleep is one of the main physiological processes affected by the COVID-19 pandemic. Some studies observed a decrease in self-reported sleep quality [15–17] and higher rates of sleep problems [18–22], although increases in time in bed [16, 23] and sleep duration [15, 23] were also reported. According to these studies, increased stress from life changes or increased psychological health issues might have negatively influenced sleep quality despite increased total time slept. Similarly, other studies found an increase in perceived stress and anxiety levels associated with worse sleep quality, more frequent nightly awakenings [24], shorter sleep duration, prolonged sleep latency, and increasingly irregular sleep rhythms [25, 26]. Robillard and colleagues (2020) [27] reported reduced time in bed and delayed sleep in a sample of more than 5,000 Canadian respondents. Worse sleep quality was also associated with lower socioeconomic resources [26]. In a sample of 217 Italian participants, Alfonsi et al. (2021) [28] found longer sleep latency, worse sleep efficiency, massive sleep medication use, but also increases in sleep duration and better daytime functioning during the lockdown. Finally, Salfi et al. (2021) [29] observed that women showed the worst condition for perceived stress, sleep quality, anxiety, insomnia, and depression symptoms, but also seemed to show greater long-term resilience during the lockdown. On the other hand, male participants showed a gradual worsening of perceived stress, sleep quality, and insomnia symptoms.

Regarding dreams, some studies also found a higher frequency of nightmares during the pandemic [25, 30, 31]. Pandemic dreams tended to reflect mental suffering, fear of contagion, and important changes in daily habits [25, 32, 33] with people who were most strongly affected by the pandemic reporting the strongest effects on their dream life, including heightened dream recall frequency [34–37]. Similarly, an increased nightmare recall frequency was associated with higher levels of perceived stress [25] and some studies indicate a possible association with a higher risk of suicide [38]. Regarding only the second wave of the pandemic, Scarpelli and colleagues found that the tone of dreams was more negative than the first wave [39] and that nightmares were more frequent in those who tested positive for COVID-19 when compared with those who were not infected by the coronavirus [40]. According to Iorio et al. [34], the tensions caused by COVID-19 resulted in a remarkable presence of dreams with emotional intensity, showing more negative emotions and sensory impressions. In addition, a study also showed that the negative impacts caused by the COVID-19 pandemic tend to be more expressed in the dreams of women and people with higher levels of education [35].

Despite the growing body of knowledge regarding pandemic-related changes in sleep and dreaming, to date, there is no quantitative study focused on the investigation of lucid dreaming, a dream state during which subjects know that they are dreaming [41, 42]. To close this gap, we set out to explore lucid dreaming recall frequency using an online questionnaire. We hypothesize that lucid dreaming frequency increased during the pandemic and that this would be associated with an increase in dream recall frequency, nightmares, anxiety, sleep fragmentation, and sleep extension.

## 2 Materials and methods

### 2.1 Participants

The study enrolled a convenience sample of 1,903 respondents from all the 26 Brazilian states and the Federal District. Participants had access to the survey through university communication channels, links on social media, and email groups, during the period from May to June 2020. We excluded from the statistical analysis underage (<18 years old) volunteers (n = 35; 1,84%) and those participants who classified their genders as other than male or female (n = 11; 0.6%), due to the very small sample size of this particular cohort. Thus, the final sample consisted of 1,857 respondents (75% women and 25% men; age mean = 32,7 +/- 12,1 years; S1 Fig in S1 File). Participation in the study was voluntary and unpaid. All respondents completed a term of consent, which described in detail the study and provided ethical permission. The need for consent was waived by the ethics committee of Brain Institute—the Federal University of Rio Grande do Norte, Natal–Brazil, and the study was conducted according to the principles expressed in the Declaration of Helsinki.

### 2.2 Questionnaire

We used the International COVID Sleep Study Questionnaire (ICOSS) [43], which aimed to understand the possible effects of COVID-19 on sleep, dream, and wakefulness. The questionnaire included the following sections: socio-demographic data (e.g. state of residence, age, gender, level of education, race, and ethnicity); pandemic and COVID-19 (e.g. was financial status affected by the pandemic?; did the respondent have COVID-19?; was the respondent living with other people during the period of social isolation?); psychological aspects (e.g. anxiety and depression); habits and general health (e.g. smoking and drinking habits; diagnosed diseases); sleep (e.g. at what time the respondent usually falls asleep?; how is the quality of sleep?); dreams (e.g. with which frequency the respondent remembers dreams); sleep and dreams before and during the pandemic (e.g. how often the respondent has nightmares currently vs. during the pandemic?). An additional question about lucid dreams was included in the Brazilian version of the questionnaire, which asked about people’s frequency of lucid dreaming recall before and during the pandemic.

The questionnaires used to assess psychological dimensions were: *Generalized Anxiety Disorder-2* (GAD-2) [44]; *Patient Health Questionnaire-2* (PHQ-2) [44]; *Stress* [45]; *Post-traumatic stress disorder* [46]; *Well-Being Index* (WHO-5) [47]; and *Quality of life and health* [48]. The questionnaires used to assess sleep and dream variables were: *Nordic sleep questionnaire* [49] *Insomnia Severity Index* (ISI) [50]; *The Snoring*, *Tiredness*, *Observed apnea*, *high blood Pressure (STOP) questionnaire* [51]; and *REM sleep behavior disorder* [52].

### 2.3 Descriptive analysis

The descriptive analysis was performed using RStudio, and graphics were created from correlation tests. The variables were codified for the Likert scale, and those analyzed to verify a possible correlation with the lucid dream recall frequency were divided into three types: 1) Sociodemographic variables: gender (male; female), age (>18 y), marital status, number of residents in the house, among others; 2) Pandemic variables: stress, sleep extension, anxiety, etc.; 3) Sleep variables: dream recall frequency, nightmare recall frequency, REM sleep behavior disorder symptoms, sleep quality, and others.

### 2.4 Pre-processing

At this stage, redundant information was pruned out from the dataset according to the distance in a hierarchical clustering of variables. Whenever the similarity between a pair of variables turned out to be too high, the variable with lower general polychoric correlations with the remaining variables was kept in the dataset, whereas the other was excluded. The amount of missing data was used as an additional criterion to filter redundant information, except when deductive replacement of non-available information was possible. The question addressing the occurrence of diseases was segregated into individual binary variables for each disorder of interest. In addition, the maximum number of residents in the same household was truncated at 10.

To investigate the effect of the COVID-19 pandemic, we dichotomize the frequency of lucid dreams into two categories: 1 –Low frequency (people reporting lucid dreaming less than monthly); and 2 –High Frequency (at least monthly, i.e., the union of categories “less than weekly”, “1–2 times a week”, “3–5 times a week” and “almost every night”), according to the Gackenbach criterion [53]. In turn, when the analysis aimed to test the factors influencing the change in lucid dreams frequency along with the pandemic, the response reflected whether the recurrence of lucid dreams increased or not during the pandemic, as compared to before COVID-19 had emerged.

### 2.5 Statistical analysis

To test the effect of the pandemic on the frequency of lucid dream recall, the recurrence of these oneiric events was modeled by a mixed logistic regression model as a function of the pandemic while controlling for relevant demographic predictors. In this context, the respondents were considered the random effect, being an individual intercept fitted to each participant. For the analysis of the factors influencing an increase in the frequency of lucid dreams during the pandemic, we fitted a logistic regression model to predict lucid dreams frequency enhancement in terms of characteristics and events related to sleep. The statistical significance of the coefficients from the model describing each effect was tested by the Wald test, whose significance level was kept at 0.05. The presented models, which provide statistical grounds for the inference of effects, were validated by diagnostic measures, confirmatory and residual analysis. All statistical analysis was performed in R software.

## 3 Results

### 3.1 Sociodemographic characterization of the sampled population

The analyses were based on the 1,857 subjects who voluntarily replied to the online questionnaire and met eligibility criteria. The demographic profile of the sample is shown in Table 1.

**Table 1:** Demographic profile of the final sample.

Country	States	Area Classification	Gender	Age	Education	Marital Status:
Brazil: 1838	Central West: 63	Rural: 49	Male: 464	Min.: 18	Elementary School: 5	Single: 1100
Other: 19	Northeast: 1297	Urban: 1808	Female: 1393	1st Qu.: 23	High School: 476	Married: 633
	South: 114			Median: 29	Technical Education: 151	Separated: 107
	Southeast: 383			Mean: 32.67	Higher Education: 848	Widowed: 17
				3rd Qu.: 39	Master’s Degree: 285	
				Max.: 80	Doctorate: 92	

### 3.2 Descriptive analysis of lucid dreaming recall frequency

**Fig 1:**
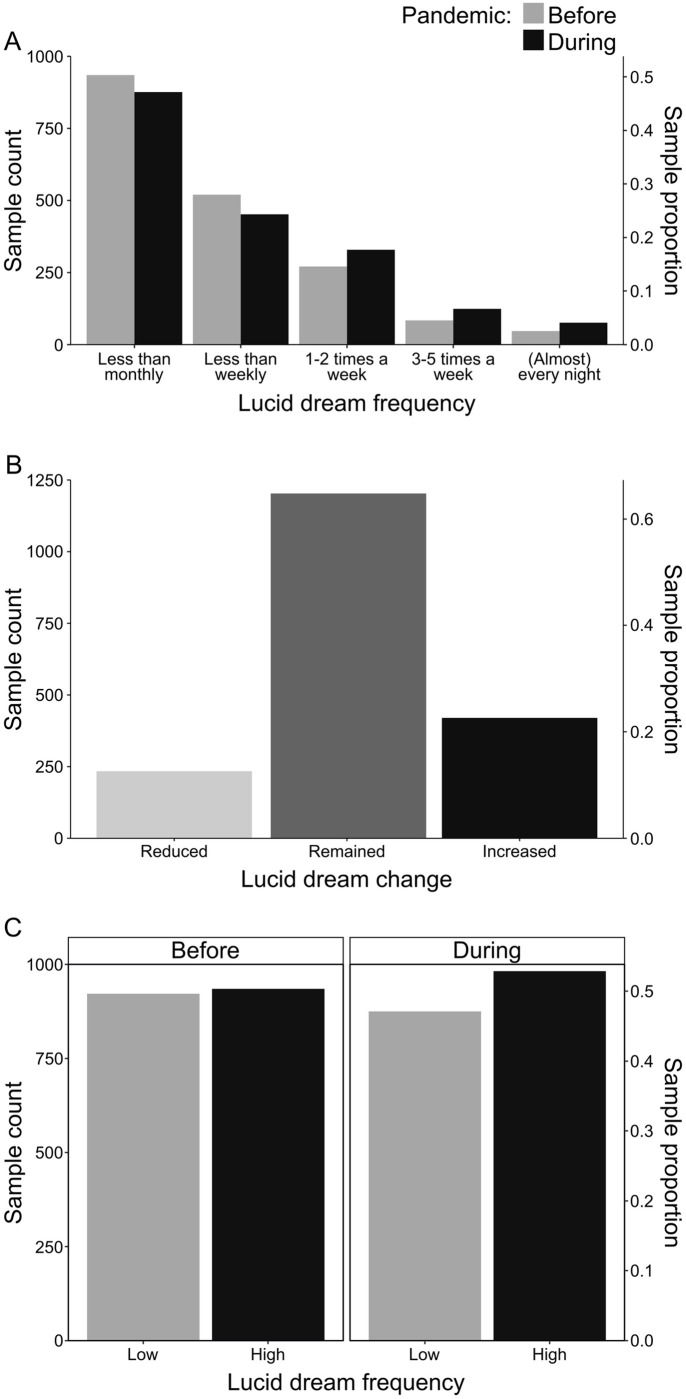
Descriptive analysis of the reported frequency of lucid dreams before and during the COVID-19 pandemic. (A) The number of subjects decreases as the frequency of lucid dream recall increases, but the number of frequent lucid dreamers increased during the pandemic. (B) Most participants maintained the level of declared frequency of lucid dreams despite the pandemic, but a considerable fraction of them informed that lucid dreams became more frequent. (C) When using the Gackenbach criterion for low and high frequencies of lucid dreaming, it is notable that the proportion of frequent and infrequent lucid dreamers is almost the same, and that the percentage of frequent lucid dreamers is discretely higher during the pandemic.

As the main substratum for the present work, we report the frequency of lucid dreams in Fig 1. Accordingly, the number of respondents declaring to have lucid dreaming progressively decreased according to its frequency, i.e., whereas the majority declared to have lucid dreaming less than monthly, few subjects declared to have lucid dreaming almost every night (Fig 1A). That is, the number of people declaring to have lucid dreaming almost every night (47 before and 76 during the pandemic) was much smaller than the number of people declaring to have lucid dreaming less than once a month (935 before and 876 during the pandemic). This analysis evidences the rarity of the phenomenon of lucid dreaming. However, a different scenario appears when using the Gackenbach criterion [53] of classification of low and high frequency of lucid dreams. According to this criterion, we dichotomized the data into two categories: 1 –Low frequency (people having lucid dreaming less than monthly); and 2 –High Frequency (at least monthly, i.e., the union of categories “less than weekly”, “1–2 times a week”, “3–5 times a week” and “almost every night”). With this approach, we observed that the number of high-frequency lucid dreamers before the pandemic (50,4%) is almost the same and discretely superior compared to low-frequency lucid dreamers before the pandemic (49,6%) (Fig 1C).

Besides that, in contrast with remembrance before the pandemic, the total of participants reporting having lucid dreams at least once per week increased during the pandemic (Fig 1A). In line with this, a considerable fraction (22.62%) of them informed that lucid dreams became more frequent, although most participants (64.78%) maintained the level of declared frequency of lucid dreams, and a smaller subset (12.60%) reported rather a mitigation of such oneiric events during the COVID-19 pandemic (Fig 1B). It is worth noting that the correlation structure (in compliance with the repeated measure nature of the data) does not take into account this qualitative exploratory analysis. Thus, at least in qualitative terms, it sounds reasonable to hypothesize that the pandemic increased lucid dreams recall frequency. This was confirmed when we used the Gackenbach criterion [53] of classification of low and high frequency of lucid dreams, as shown in Fig 1C: the percentage of frequent lucid dreamers is discretely higher during the pandemic (52,8%), compared to before the pandemic (50,4%).

### 3.3 The effect of the pandemic on the frequency of lucid dreams

**Fig 2:**
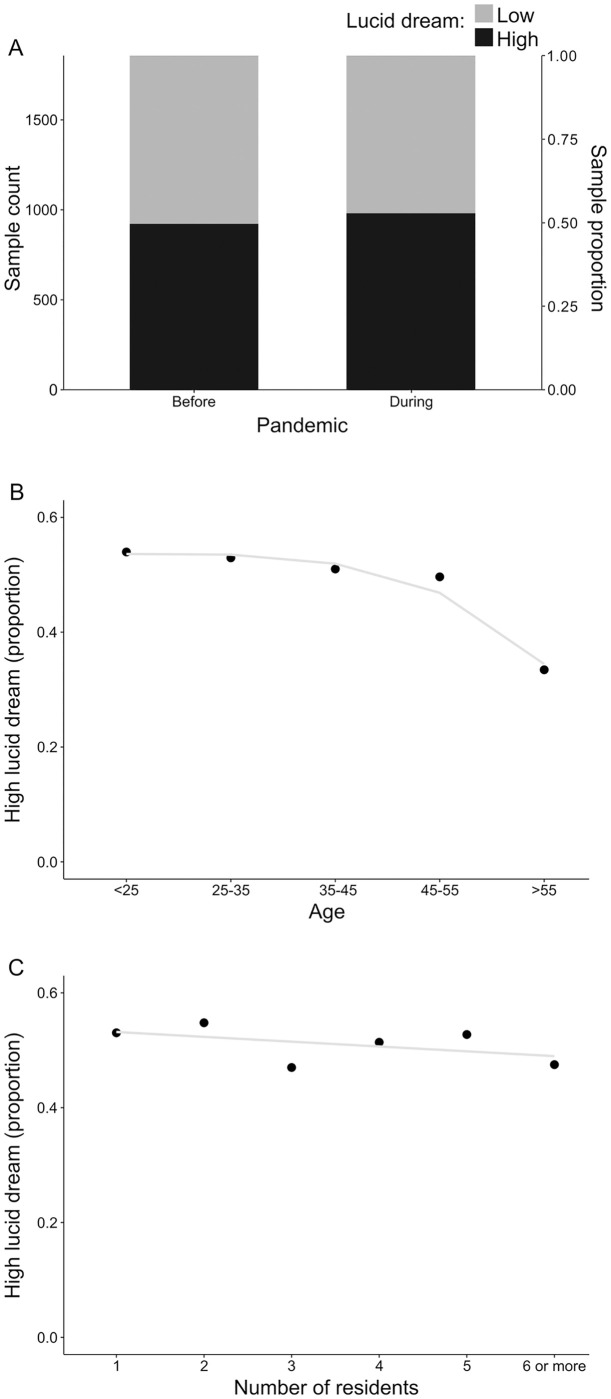
Representation of main effects unveiled by the mixed logistic statistical model. (A) The proportion of high lucid dreamers before and after the pandemic. (B) The proportion of respondents declaring high frequency of lucid dreams according to the corresponding category for age. Because these categories are somewhat arbitrary, a rough smoothing for the relation between the variables (blue line) was added to the plot to improve interpretation. (C) The proportion of high lucid dreamers conditioned to the number of residents sharing the house. In the absence of any clearer representation, the relationship between these variables is summarized by a straight (blue) line to enhance visual inspection.

To test this hypothesis, we fitted a mixed logistic regression model that could account for the putative effect of the pandemic on the frequency of lucid dreams while controlling for (otherwise) confounding variables of demographic nature. Such statistical modeling was supported by descriptive analysis (Fig 1) and, for this purpose, the recurrence of lucid dreams was measured in a binary scale: low (less than monthly) or high (more than monthly). In this context, the effect of pandemic on lucid dreams recall frequency was found to be statistically significant (Table 2; Wald Test: estimate = 0.3031, std error = 0.1019, z value = 2.976, p = 0.00292). In other words, the proportion of subjects recalling their lucid dreams more often (more than monthly) was found to increase during the pandemic, being such an increment supported by its statistical significance. Accordingly, the strength of the association between pandemic and lucid dreams frequency could be measured by an odds ratio equal to 1.3541, indicating that the odds for high frequency of lucid dreams during the pandemic were estimated as 35.41% higher than equivalent odds before COVID-19. This scenario emerges as a consequence of a modest (although significant) increase in the proportion of respondents with high lucid dreams frequency during the pandemic as compared to before it (Fig 2A). Once only demographic predictors were controlled, such an association measure can represent a quantification of the total effect of the pandemic on lucid dreams frequency.

**Table 2:** The Wald test for the coefficients embodying the mixed logistic regression model to account for the effect of the pandemic on lucid dreams while controlling for age and number of residents.

Wald test				
	Estimate	Std. error	z value	p
**(Intercept)**	1.9993	0.4273	4.679	0.00000289
**Pandemic**	0.3031	0.1019	2.976	0.00292
**Age**	-0.0448	0.0090	-4.974	0.00000066
**Residents**	-0.1745	0.0748	-2.332	0.01971

On modeling the effect of the pandemic, the only demographic predictors with enough influence on the frequency of lucid dreams to demand being controlled were respondents’ age and the household size (in terms of the number of residents). In this context, the impact of age on the frequency of lucid dreams was found to be statistically significant (Table 2; Wald Test: estimate = -0.0448, std.error = 0.0090, z value = -4.974, p = 0.00000066). Accordingly, when other predictors were fixed, the odds for high frequency of lucid dreams reduced 4.38% for every one year of age increment (odds ratio = 0.9562). The magnitude of such an effect is depicted in Fig 2B in terms of the proportion of high lucid dreamers. In addition, the influence of the household size (in terms of number of residents) on lucid dreams frequency was also statistically significant (Table 2; Wald Test: estimate = -0.1745, std.error = 0.0748, z value = -2.332, p = 0.01971). In this case, while other predictors are kept constant, as compared to low frequency of lucid dreams, the odds for high recurrence of these oneiric events decrease 16.01% for every unit increase in the household (odds ratio = 0.8399). This effect is represented in Fig 2C, suggesting a subtle decrease in the proportion of high lucid dreamers as the number of residents increases.

Other demographic predictors, such as ethnic group, gender, and perception of economic loss because of the pandemic, lacked enough evidence to endorse their statistical significance, hence were pruned out from the statistical model for the sake of parsimony. In turn, education and marital status could not be jointly tested along with age because of considerable correlations among these variables (S2 Fig in S1 File). At last, the effect of the respondents (i.e., the random component of the statistical model) was estimated to vary in the population according to a standard deviation equal to 3.256. The statistical model substantiating the inference was validated by residual analysis (S3 Fig in S1 File).

### 3.4 Sleep variables influencing the increase in lucid dreams frequency

After corroborating the statistical significance of the association between the pandemic and the frequency of lucid dreams, we investigated factors that could help to elucidate the increment of these oneiric events. Besides the information from exploratory analysis (S4 Fig in S1 File), we used AIC (Akaike information criteria) based stepwise to select the predictors embodying a logistic regression model to predict the probability of increased frequency of lucid dreams during the pandemic. Pairs of predictors with short distance (on hierarchical clustering, S5 Fig in S1 File) or substantial polychoric correlations were avoided to protect the inference against bias from multicollinearity.

**Fig 3:**
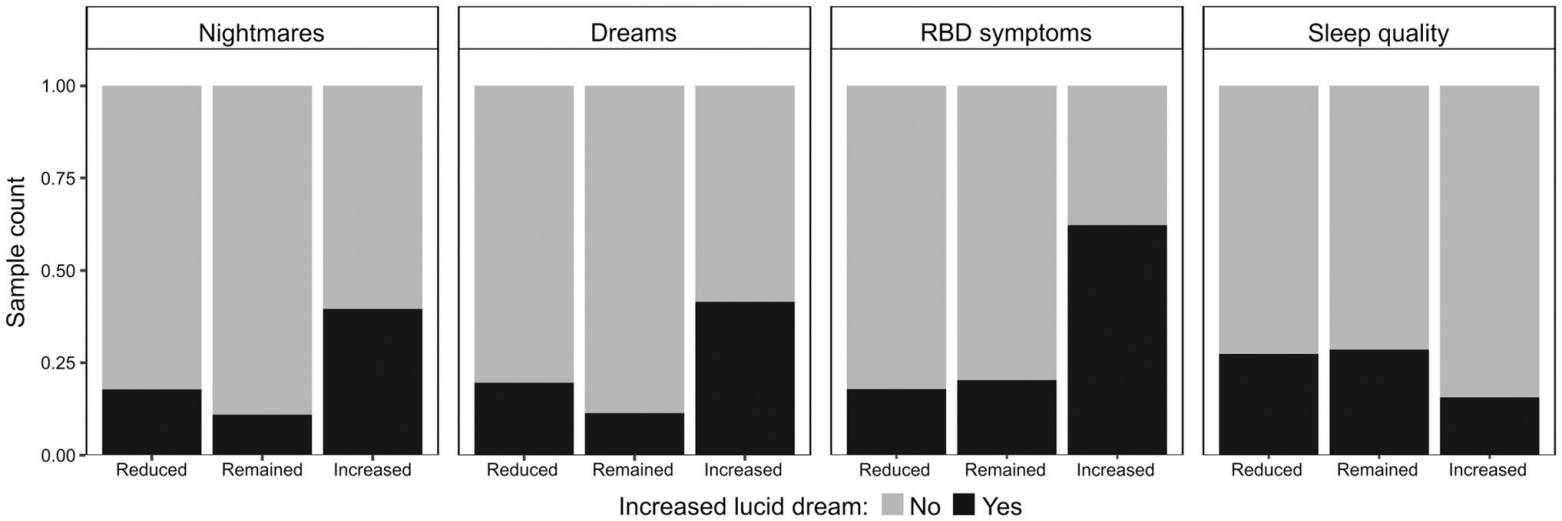
Representation of the associations unveiled by the logistic regression model. For each predictor used to embody such a model (A = nightmares, B = dreams, REM sleep behavior disorder symptom, D = sleep quality), the association is represented by the differences (among corresponding predictor’s levels) regarding the proportion of respondents declaring increased frequency of lucid dreams during the pandemic.

The resulting model has its coefficients exhibited in Table 3. Accordingly, the increase in the recurrence of lucid dreams during the pandemic was found to be associated with an increment in the frequency of remembering nightmares (Table 3; Wald test: estimate = 1.1877, std. error = 0.1384, z value = 8.584, p < 2e-16). For the respondents who remembered less than one nightmare a month, 10.81% of them experienced an increment in lucid dreams frequency. This proportion progressively increased as remembering nightmares became more usual, achieving a maximum of 41.62% of the participants, whose nightmares were remembered from three to five times a week. Among subjects declaring (almost) daily frequency of nightmares, the proportion that experienced an increase in the frequency of lucid dreams was slightly lower, 37.78%. The strength of such association measured by an odds ratio is equal to 3.2795. In this context, the odds for an increase in lucid dreams frequency were 227,95% higher when the recurrence of nightmares also enhanced during the pandemic as compared to the odds among respondents whose frequency of nightmares became stable for the same period. This association is illustrated in Fig 3A, which indicates that the proportion of respondents declaring increased frequency of lucid dreams was substantially greater (3.62 times higher) among those that also experienced an increment in remembering nightmares as compared to peers whose frequency of nightmares remained stable during the pandemic. The association between increased recurrence of lucid dreams and nightmare frequency lacked enough evidence to endorse its statistical significance when respondents declaring a reduction in nightmares frequency were compared to those reporting their stability during the pandemic (Table 3; Wald test: estimate = 0.3309, std. error = 0.2805, z value = 1.180, p = 0.23807).

**Table 3:** The Wald test for the coefficients embodying the logistic regression model to account for the influence of sleep events on the probability of an increased frequency of lucid dreams during the pandemic. For every predictor, the neutral level was used as a reference.

Wald test				
	Estimate	Std. error	z value	p
**(Intercept)**	-2.2557	0.1597	-14.129	< 2e-16
**Nightmares: reduced**	0.3309	0.2805	1.180	0.23807
**Nightmares: increased**	1.1877	0.1384	8.584	< 2e-16
**Dreams: reduced**	0.3046	0.1985	1.534	0.12503
**Dreams: increased**	1.3511	0.1388	9.38	< 2e-16
**Singing while asleep: reduced**	-0.3664	0.3768	-0.972	0.33083
**Singing while asleep: increased**	1.2010	0.2288	5.249	1.53e-07
**Sleep quality: poor**	-0.2532	0.1564	-1.619	0.10540
**Sleep quality: good**	-0.5064	0.1596	-3.173	0.00151

In addition, an increment in the frequency of remembering dreams during the pandemic was also found to be associated with an increase in the recurrence of lucid dreams in the same period (Table 3; Wald test: estimate = 1.3511, std. error = 0.1388, z value = 9.38, p < 2e-16). Among participants reporting remembering less than one dream a month, 9.35% of them experienced an increase in lucid dreams frequency during the pandemic. Such a proportion mounted up, achieving 32.45% of those who remembered three to five dreams a week, then reducing to 26.50% for participants remembering dreams (almost) daily. Such an association was characterized by an odds ratio equal to 3.8617, indicating that the odds for an increased lucid dreams frequency were 286.17% higher in cases where remembering dreams became more usual during the pandemic as compared to those subjects whose frequency of recalling dreams remained unchanged. On the other hand, a reduction in the frequency that dreams were remembered during the pandemic seems to have no statistically significant consequence to the probability of increasing the recurrence of lucid dreams (Table 3; Wald test: estimate = 0.3046, std. error = 0.1985, z value = 1.534, p = 0.12503). As a result, when compared to participants whose remembrance of dreams remained constant during the pandemic, the proportion of respondents declaring increased lucid dreams frequency was considerably (3.64 times) higher among those who also experienced an increment in remembering dreams in that period (Fig 3B). In turn, the difference for such a proportion was much less expressive between the sets of respondents with a reduced and stable frequency of remembering dreams along with the pandemic.

Likewise, we could only find enough evidence to corroborate an association between an increase in lucid dreams frequency and the recurrence of singing during sleep in cases that this behavior increased during pandemic (Table 3; Wald test: estimate = 1.2010, std. error = 0.2288, z value = 5.249, p = 1.57e-07), but not when the frequency of sleep singing reduced (Table 3; Wald test: estimate = -0.3664, std. error = 0.3768, z value = -0.972, p = 0.33083). Thus, we have no evidence to consider that the odds ratio would depart from the unit when the respondents experiencing a reduction in the frequency of sleep singing were contrasted with those who declared stability for such behavior during the pandemic, in terms of the probability of an increased recurrence of lucid dreams. In turn, the odds for an enhanced lucid dreams frequency were found to be 232.34% higher in cases in which the frequency of singing while sleeping increased during the pandemic as compared to peers who did not experience changes in this behavior for the same period (odds ratio = 3.3234). Such an association is reflected in Fig 3C, which indicates that the proportion of respondents declaring increased frequency of lucid dreams during the pandemic was much (about 3.07 times) higher among those who also experienced an increment in the frequency of sleep singing.

Lastly, sleep quality was also found to be significantly associated with lucid dreams frequency. However, such an association occurs when good sleep is compared to neutral quality (Table 3; Wald test: estimate = -0.5064, std. error = 0.1596, z value = -3.173, p = 0.00151). In this context, it is worth noting that the five-point scale used to acquire the quality of sleep was compressed into a three points scale while preserving for the neutral point. Thus, the categories for poor and considerably poor sleep were collapsed as poor in the same way that good and considerably good were collapsed as good. Under these circumstances, the odds for an increased frequency of lucid dreams were 39.73% lower among respondents declaring a good sleep during the pandemic than corresponding odds from subjects reporting a neutral sleep for the same period (odds ratio = 0.6026613). In turn, when compared to the neutral quality, any consequence of poor sleep was devoid of evidence to endorse its statistical significance (Table 3; Wald test: estimate = -0.2532, std. error = 0.1564, z value = -1.619, p = 0.10540), hence keeping the corresponding odds ratio as a unit. This scenario is illustrated in Fig 3D, where differences regarding the proportion of respondents declaring increased frequency of lucid dreams become noticeable only when other categories for sleep quality are contrasted to good sleep (about 45% reduction).

When all these predictors (changes in the frequency of recalling nightmares, remembering dreams, singing during sleep, and sleep quality during the pandemic) were controlled, respondent’s age and the number of residents lacked enough evidence to support their statistical significance, hence were removed from the model for the sake of parsimony. Needless to say, as compared to the model introduced in Table 2, the model described in Table 3 addresses a different hypothesis concerning a different aspect of the relation between the pandemic and lucid dreams. Thus, it is not surprising that different predictors were found to be relevant for these models. In addition, statistical models must be appreciated in the context of the limited information provided by the sample. Under these circumstances, each model aims to make the best use of available information to incorporate the predictors with the most prominent effect so as to describe them, because such predictors contribute most to the understanding of the phenomenon. In this regard, when a predictor is not included in a model, it does not imply that it has no effect, but rather it means that its effect is smaller (relative to the natural variability) and, after identifying the big effects, the evidence provided by the sample turned out to be insufficient to endorse its statistical significance. Finally, it is worth noting that the statistical model (introduced in Table 3) was further validated according to diagnostic measures (S7 Fig in S1 File), confirmatory (S1 Table in S1 File), and residual analyses (S8 Fig in S1 File), thus endorsing the inferences derived from it.

## 4 Discussion

We observed that a considerable fraction of the subjects informed that lucid dreams became more frequent during the pandemic. In line with that, the total of participants who report having lucid dreams at least once per week increased during the pandemic. Using a mixed logistic regression model, we confirmed that the COVID-19 pandemic increased significantly lucid dreaming recall frequency. We also observed that this increase in lucid dreaming during the pandemic was significantly associated with an enhancement in both dream and nightmare recall frequencies, as well as with sleep quality and symptoms of REM sleep behavior disorder. To our knowledge, this is the first consistent report that focused on lucid dreaming during the COVID-19 pandemic.

### 4.1 Lucid dreaming recall frequency

As expected, the number of respondents declaring to have lucid dreaming progressively decreased according to frequency. That is, the number of people declaring to have lucid dreaming almost every night was much smaller than the number of people declaring to have lucid dreaming less than once a month (Fig 1A). A similar pattern of lucid dreaming recall frequency was observed in a German sample [54] and in a meta-analysis conducted by Saunders et al. [55], in which they suggest that 55% of the world population had a lucid dreaming episode at least once in a lifetime, while only 23% reported experiencing lucid dreaming at least once a month. It is essential to note that the scales used in these studies are different from the scale used here. In our scale, the lower frequency considered, “less than monthly”, represents a large window, because it does not allow us to distinguish clearly between rare lucid dreamers (e.g.: a lucid dream episode per year, or two on a lifetime) and moderate lucid dreamers (e.g.: a lucid dream every two or three months). Despite that, it is still clear that the number of subjects decreases as the frequency of lucid dream recall increases (Fig 1A).

Nevertheless, using the Gackenbach criterion [53] of classification of low and high frequency of lucid dreams (Fig 1C), we observed that the number of high frequency lucid dreamers (50,4%) before the pandemic is almost the same as low-frequency lucid dreamers (49,6%) before the pandemic. Thus, the number of high-frequency lucid dreamers in our study is much superior to the expected incidence of 23% reported by Saunders et al. [55]. In these meta-analyses, only one study presented a prevalence of frequent lucid dreamers higher than 32%, which is Stumbrys et al. [56], who found a prevalence of 49,8%. In a previous study in a Brazilian sample, we observed that about 16% of participants experience lucid dreaming every week or almost every day [57].

We consider two main hypotheses to explain the high incidence of frequent lucid dreamers found in our present study. First, our sample is mainly constituted of young women, who comprise the cohort with higher dream recall frequency, which is closely associated with high lucid dreaming recall frequency [58]. Women are also more prone to report dreams and tend to be more interested in dreams than men [59]. Second, since our respondents had access to the survey through university communication channels, social media, and email groups, the recruitment method could have biased our sample since the people who agreed to participate are probably interested in dreams and lucid dreaming, and it is known that people more interested in dreams also tend to have more lucid dreams [60].

### 4.2 The pandemic effect

We found that the COVID-19 pandemic increased significantly lucid dreaming recall frequency. On modeling the effects of the pandemic, the only demographic predictors with enough influence on the frequency of lucid dreams to demand being controlled were respondent age and the household size, in terms of the number of residents, both showing to be statistically significant on the influence over the frequency of lucid dreams (Table 2, Fig 2).

As far as we know, there are no previous consistent studies focused on the investigation of lucid dreams during the pandemic. Nevertheless, Scarpelli et al. [24] investigated the impact of the end of COVID confinement on dreams in an Italian sample and found an increase in lucid dreaming frequency during the pandemic, compared to the end of the lockdown. Scarpelli et al. [61] also investigated the dream activity in a sample of narcoleptic patients in Italy, during the COVID-19 lockdown, and found higher lucid dreaming frequency compared to controls. However, the authors found no evidence of the influence of the pandemic on these changes in lucid dreaming frequency.

There are many elements in the COVID-19 pandemic that could be influencing this increase in lucid dreaming. The pandemic has been a period characterized by social isolation, fear of one’s own death, and fear of losing family and friends, thus, being characterized as a period of increased stress rates [5–7]. Studies conducted during the pandemic suggest that the higher levels of stress and tensions had a major influence on patterns of sleep and dreaming [32, 33], including heightened dream recall frequency [34, 35, 61], increased nightmare frequency [25] and more intense and negative dreams [41].

Bottary et al. [62] reported anecdotal evidence regarding an increase in dream recall frequency and more vivid dreams during the pandemic and hypothesized that it may be attributable to increased sleep fragmentation caused by the sleep extension many people are experiencing. A recent study identified that 73.9% of people with low levels of stress during the lockdown experienced an increase in the duration of sleep when compared with the pre-pandemic period [25]. Some studies, however, bring results contrary to those obtained so far. Brand et al. [63], for example, found that increased dream recall frequency was independently predicted by factors such as female gender, sleep quality, and creativity, whereas perceived stress, awakenings during the night, and sleep duration had no predictive value.

We previously observed, also in a Brazilian sample, but not in a pandemic scenario, that stress and sleep with no pre-determined time to wake up were pointed out for the subjects as some of the main factors to influence the occurrence of lucid dreaming [57]. This could be explained by an increase in the awakenings from REM sleep that is associated with stress [64]. Due to the lockdowns and consequent interruption of school classes and the adoption of a homework schedule, some people are staying more time at home, and thus sleeping more [16, 23]. This may have increased the duration of REM sleep, which is the sleep stage more associated with lucid dreams [42, 43], despite lucid dreams can also happen during N1 and N2 sleep stages [65]. Thus, stress and sleep extension likely explain the increase of lucid dreaming frequency during the COVID-19 pandemic. In the model presented here, however, stress and total time slept had no statistical significance over the increase of the frequency of lucid dreams. Despite that, there is a possibility that stress and total time slept are interfering with the frequency of lucid dreams indirectly, through the influence on sleep variables such as dream and nightmare recall frequencies, and sleep quality. Nevertheless, there is no concrete data to sustain the hypothesis of an indirect association between stress, time slept, and lucid dreaming recall frequency.

Time of quarantine and knowing someone with COVID-19 also seem to have no association with the frequency of lucid dreams. The number of residents in the same household correlated negatively with lucid dream frequency, but this seems to have no significant influence on the role of the pandemic over the increase of lucid dream frequency, once the sleep variables were controlled. Given the circumstances, it remains unclear which particular pandemic factors interfere with the increase in the frequency of lucid dreams. The variables that were significantly associated with the increase in the frequency of lucid dreams will be discussed below.

### 4.3 Factors influencing lucid dream recall frequency: Sleep variables as a bridge for the pandemic effect

The following variables showed an association with the increase of lucid dreaming recall frequency during the pandemic: dream recall frequency, nightmare recall frequency, singing during sleep (as a possible symptom of REM sleep behavior disorder), and sleep quality (Table 3, Fig 3). It is important to look at these factors carefully, in order to try to explain their associations with lucid dream recall frequency, once they are also closely related, and exert a complex (even not well-known) relation to each other.

We expected that dream and nightmare recall frequencies would be the main factors (from the dataset) influencing the probability of an increment in lucid dreaming recall frequency during the pandemic. This expectation is reinforced by hierarchical clustering of variables, showing that (along with respondent’s age and the number of residents) dream and nightmare recall frequencies have the shortest distances to the increase in lucid dream recall frequency (S5 Fig in S1 File). In other words, as compared to the binary scale to measure the increase in lucid dreams during the pandemic, dream and nightmare recall frequencies present the highest similarities, hence carrying more similar information and higher capacity of prediction. In the next sessions, we will discuss the influence of these sleep variables over lucid dreaming frequency, in the context of the COVID-19 pandemic.

#### 4.3.1 Dream recall frequency

In our study, we found that the increment in the dream recall frequency during the pandemic was associated with an increase in the recurrence of lucid dreams in the same period. Other studies also reported an increase in dream recall during the pandemic [34, 35, 37]. The association between dreams and lucid dreaming recall has been already largely documented, i.e., the more a person remembers dreams in general, the more they will be able to remember lucid dreams (for review see [56, 58, 60, 66, 67]. An example of this is the dream diary, which consists of keeping a daily report of the dreams one remembers having during the night [68]. The dream diary is an instrument primarily projected to increase dream recall and became one of the most used and most efficient methods to induce lucid dreams. In accordance with that, Aspy [69] reported a significant increase in lucid dreaming frequency in the one-week diary compared to the participants’ retrospectively estimated lucid dreaming frequency, i.e., focusing on dreams not only increased dream recall, but also lucid dreaming recall.

However, studies suggest that the use of a dream diary only helps to increase lucid dreaming frequency significantly if the person intends to enhance lucid dreaming [69, 70], i.e., an increase in dream recall frequency alone is not sufficient to increase lucid dreaming frequency significantly—the intention to have a lucid dream is also essential. In that manner, we can say that higher dream recall frequency may be a sign of higher interest in the dream world. People who are more interested in their own dreams may spend more time trying to remember them [71, 72], including lucid dreams. Once our study counted with volunteers who spontaneously agreed in participating in the survey that they found online, there is a possibility that our sample is self-selected for people who are interested in dreams, which explains in part the association between dream and lucid dream recall frequencies found in the present study.

At last, an exploratory evaluation of our data suggests that the frequency of remembering dreams is related to the recurrence of lucid dreams. Especially during the pandemic, the proportion of respondents who declared an increment in lucid dreams frequency gradually increased as remembering the dreams became more usual. Among participants reporting remembering less than one dream a month, 9.35% of them experienced an increase in lucid dreams frequency during the pandemic. Such a proportion mounted up, achieving 32.45% of those who remembered three to five dreams a week, then reducing to 26.50% for participants remembering dreams (almost) daily. Furthermore, our sample was mainly constituted of women, who are the public with higher dream recall [41, 58, 73–76] mainly when passing through stress [77]. However, in the present study, gender lacked enough evidence to endorse its statistical significance regarding the increase of lucid dreaming during the pandemic.

#### 4.3.2 Nightmare recall frequency

We found a significant association between the increase of nightmare recall frequency and lucid dreaming recall frequency during the pandemic. The association between those variables is already known [78–81], and their relationship could be intermediated by dream recall frequency [81]. Nightmares may even trigger lucidity [82, 83], that is, when subjects are experiencing a nightmare, they try to figure a way out of it, and the thought “this is just a dream” is a relieving strategy to get through a nightmare, once it consists of realizing they are dreaming [81, 84]. Besides that, heightened physiological arousal during sleep was reported for both nightmares [85] and lucid dreaming [86, 87]. Thus, physiological arousal seems to be an important intervenient factor between nightmares and lucid dreaming recall frequency.

Furthermore, a higher nightmare recall is associated with emotionally relevant events for the dreamer, such as stress [88–90] and trauma [91]. Certainly, the collective trauma generated by the COVID-19 pandemic influenced the increase of this phenomenon in the population. Accordingly, it was found an increased frequency of nightmares during the pandemic [92] and an increased frequency of threatening events in dreams [93]. Both studies investigated patients with PTSD whose symptoms were previously (before the pandemic) in remission, and one found the re-emergence of symptoms of PTSD [92]. Nightmares are considered one of the most frequent symptoms of PTSD, reaching up to 80% of the patients [94]. In the context of PTSD, the constant interruption of REM sleep may be associated with an increase in nightmares and thus inadequate processing of emotional experiences, since REM sleep is associated with a reduction in emotions subjective the next day [95]. It is important to note that the increase of nightmares did not necessarily include COVID-19 themes, but rather pre-existing traumas.

According to other authors, however, the contents and emotions of dreams seem to be related to the difficulties imposed by the new reality of the pandemic [32, 33, 96, 97]. In this context, some studies have shown that women respond more intensively to stressful events, tending also to incubate these events more into dreams [73, 98]. Some studies have found that during the pandemic, female participants had considerably high rates of negative feelings and emotions (such as anxiety, anger, and sadness) in their dreams [99, 100]. In a study carried out with students at the University of Toronto, there was an increase in nightmares in women during the pandemic [101]. This study drew attention to the fact that the aggressive content theme of dreams increased. Another stressful factor for women is domestic violence, which increased significantly during the pandemic [102]. Compared with a previous study [103], at a time outside the pandemic, men usually had dreams with more aggressive themes. As said before, however, in the present study, the effect of gender on the (binary) frequency of lucid dreams looks negligible.

The Threat Simulation Theory [104] proposes that dreaming prepares the dreamer for future experiences. In that way, some authors interpret the increase of nightmare recall during periods of stress as a mechanism of emotional regulation [105]. Similarly, lucid dreams have a therapeutic function, being important for treating recurrent nightmares [84], promoting resilience [106], decreasing depression symptoms [107], and contributing to psychological growth [108]. In this sense, the increase of nightmares and lucid dreams during the pandemic may have a therapeutic/emotional regulation function, helping to deal with the stress of this period. In the present study, stress did not show a significant association with the increase of lucid dreaming. However, the lack of enhancement on stress rates in the present study may be a reflex of the therapeutic role of dreams and lucid dreams.

#### 4.3.3 REM sleep behavior disorder symptoms

We observed an association between singing during sleep (as a possible manifestation of REM sleep behavior disorder) and lucid dream recall frequency. Sleep singing was quite rare for most respondents, turning the sample considerably unbalanced regarding the levels of this variable, thus eroding much of the confidence on this speculative scrutiny. Nevertheless, the proportion of respondents declaring an increase in the frequency of lucid dreams seemed to enhance according to the frequency of sleep singing during the pandemic, from 20.57% among participants who used to sing while asleep less than monthly up to 62.50% among those reporting to sing while asleep three to five times a week.

REM sleep behavior disorder is a parasomnia characterized by physically acting out dreams with vocal sounds and violent movements [109]. REM sleep behavioral events, such as vocalization and motor behavior are associated with dreaming [110], once these behaviors are a reflex of the actions of the dreamer in the dream. Both REM sleep behavior disorder and lucid dreaming [111] are considered REM sleep dissociated phenomena [112]. Thus, since singing or sleep-talking are signs that a dream is occurring, it makes sense that sleep singing is associated also with the occurrence of a lucid dream and, consequently, with higher lucid dreaming frequency, as we found in our study.

#### 4.3.4 Sleep quality

We observed that self-reported sleep quality was significantly associated with lucid dreaming recall frequency (particularly during the pandemic). Many studies reported poorer sleep quality during the pandemic [15–17, 61], but while some studies found no link between lucid dreaming and sleep quality [113, 114], others reported a significant relationship between lucid dream frequency and poor sleep quality [115]. However, this relation was explained by the association between nightmares and poor sleep quality [116, 117]. Lucid dreams were also associated with positive emotions in the morning [118, 119]. In our study, it is possible that this association between sleep quality and lucid dreams is due to the positive humor in the morning provided by the experience of having a lucid dream.

## 5 Limitations

The present study utilized self-reported measures, which are prone to bias. Besides that, as the survey was applied via the internet, there is no certainty that the subjects responded to the questions carefully and that they understood the questions completely. Other limitations include uneven distribution of women and men in our sample and a brief assessment of psychological variables. Finally, the scale used to measure lucid dreams frequency has its limitations, as the window for the low-frequency lucid dream (less than monthly) was too large and represented by only one option. New studies should consider a more balanced scale, including separated options for the low-frequency lucid dream (e.g.: “never”, “less than once a year”, “less than monthly” etc.).

## 6 Conclusions

Lucid dreaming recall frequency significantly increased during the pandemic. This increase in lucid dreaming was significantly associated with an enhancement in both dream and nightmare recall frequencies, as well as with sleep quality and sleep singing. A possible explanation for this increase in the oneiric activity in general (including dreams, lucid dreaming, and nightmares) during the COVID-19 pandemic is an increase in stress, sleep fragmentation, and sleep extension, which can increase awakening, especially for REM sleep—the sleep stage more associated with lucid dreaming, dreams, and nightmares. On the other hand, more specifically regarding lucid dreams, given that the pandemic has been a period of intense negative emotions and enhanced stress, the increase of lucid dreaming recall may appear as an important factor for the promotion of resilience [106] and decreasing of depression symptoms [107]. In this sense, the increase in lucid dreaming during the pandemic may have helped deal with pandemic-related negative impacts. More research is necessary to clarify this relationship.

## Supporting information

S1 File This file contains 8 supporting figures and their captions along with a supplementary text discussing these results. (DOCX)

S1 Data (CSV)
