# Supplementary material for: Lucid dreaming increased during the COVID-19 pandemic: An online survey
Source: PLoS One. 2022 Sep 14;17(9):e0273281. doi: 10.1371/journal.pone.0273281 (PMC9473433; doi:10.1371/journal.pone.0273281)
Supplement: S1 File — (DOCX) [file pone.0273281.s001.docx]

**Supplement Material**

Because of the inclusion criteria, we considered no respondent younger than 18 years old, which resulted in a range of values for age from 18 up to 80 years. Accordingly, one quarter of the sample was younger than 23, the modal value, and half of the respondents were no older than 29 years old. The dispersion became higher as age increased, being 39 up to 80 the range of age for the quarter of oldest subjects (Figure S1).


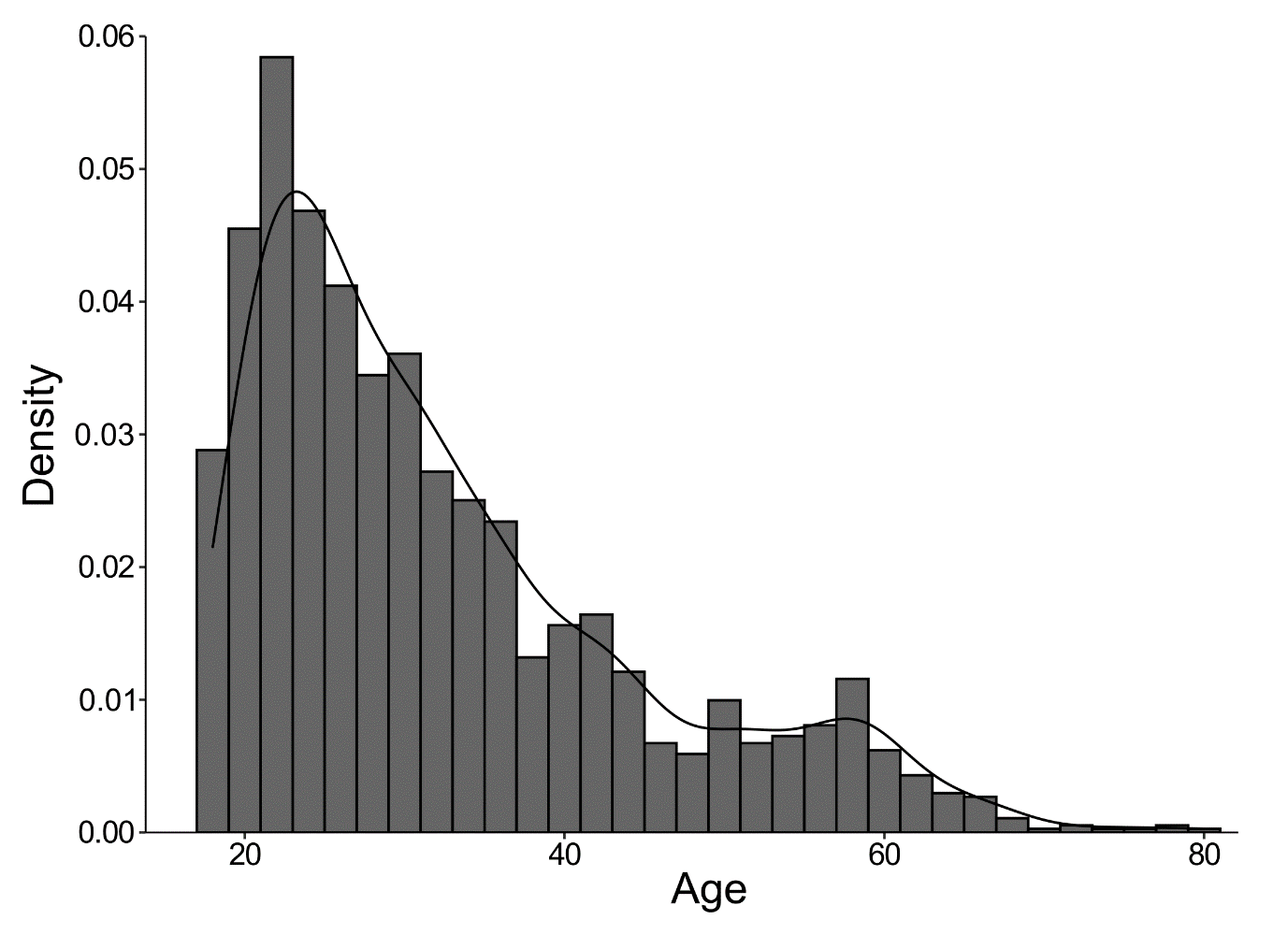


Figure S1 – Age dispersion of the sample

*The effect of pandemic on the frequency of lucid dreams*

After speculating about the marginal distribution of lucid dreams frequency (Figure 1), descriptive analysis was carried out to estimate the conditional distribution of such a response according to the levels of some demographic variables. In other words, we wondered how plausible it was to find evidence that the distribution of lucid dreams frequency (both before and during the pandemic) could be affected by these demographic predictors. At first, the proportion of respondents declaring high frequency of lucid dreams was found to progressively shrink as age increased (Figure S2-A). Despite some variability (reasonably compatible with the chance), the same pattern took shape disregarding the pandemic, except that the curve for the proportion of high lucid dreamers across ages looked shifted toward a slightly higher level during the pandemic in contrast with before it. In this sense, the putative effect of the pandemic on lucid dreams frequency seems not to depend on (interact with) age. The same picture takes shape when the household size is taken into account (Figure S2-B). In general terms, the curve for the proportion of high lucid dreamers during the pandemic according to the number of residents closely hovers over the counterpart before covid-19 had emerged. This scenario indicates that there is no clear evidence supporting that the effect of the pandemic on lucid dreams might depend on the number of residents. In turn, a putative main effect of household size on lucid dreams cannot be readily discarded, given the noticeable variability of the proportion of high lucid dreamers according to the number of residents (Figure S2-B).

**
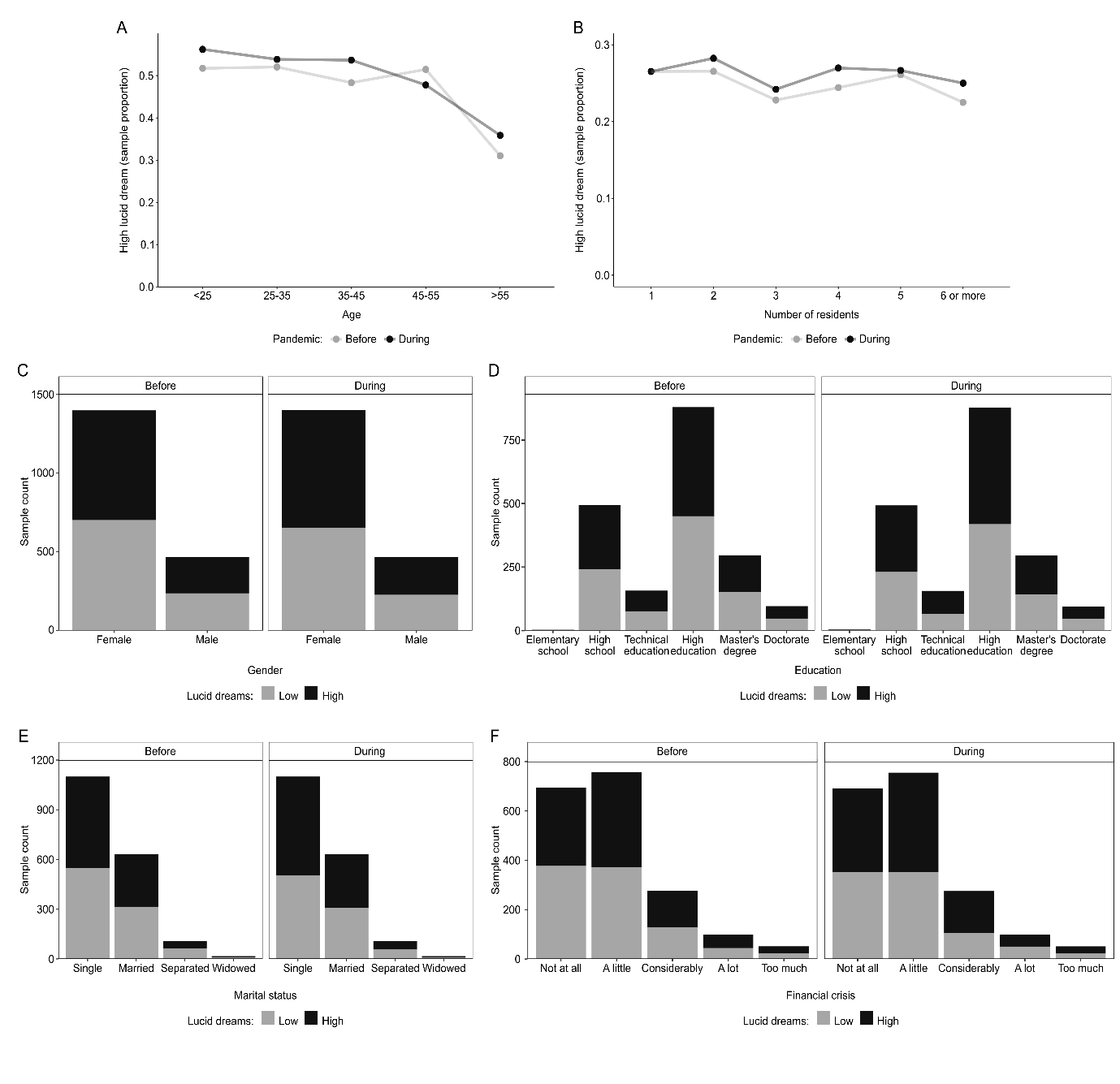
**

Figure S2 - Descriptive analysis of the sample distribution of the frequency of lucid dreams conditioned to the levels of demographic explanatory variables. In A, despite being acquired on a discrete scale, respondents’ ages were categorized into somewhat arbitrary levels to compute proportions. In B, only for representation, the number of residents was truncated at 6. From A to F, the demographic variables were assumed to remain constant along with the pandemic, thus “before” and “during” refer to the period of time that the frequency of lucid dreams was concerned relative to the covid-19 pandemic.

The frequency of lucid dreams before the pandemic recalled by respondents seems to be insensitive to the gender of the participants. In this context, virtually half of both self-declared males and females (49.14% and 49.82%, respectively) reported high frequency of lucid dreams (Figure S2-C). The scenario looked considerably stable during the pandemic when 51.23% of the men and 53.34% of the women from the sample declared a high frequency of these oneiric events. Thus, under any conceivable speculation of sampling variability, the effect of gender on the (binary) frequency of lucid dreams looks negligible, so seems to be an interaction between gender and pandemic. In other words, gender seems to have no effect on the frequency of lucid dreams, and whatever the effect the pandemic may have on this phenomenon it seems not to depend on gender.

In turn, the descriptive analysis suggests that education may modulate the effect of the pandemic on the frequency of lucid dreams. Apart from subjects with elementary education (whose sample size was too short for appraisal), all educational levels exhibited a proportion of high lucid dreamers in the range from 49% to 52% before the pandemic (Figure S2-D). In general, for these categories of education, if there was any change at all, the proportion of respondents declaring a high frequency of lucid dreams imperceptibly increased during the pandemic. The exception was the subset of respondents with technical education, whose increment was found to be slightly more substantial (6.62%) mounting 58.28% of high lucid dreamers in this educational category.

In addition, the influence of marital status on the frequency of lucid dreams does not look so evident (Figure S2-E). In terms of lucid dreams frequency, before the pandemic, the subsets of single and married respondents were quite comparable, both presenting 50% of high dreamers. The proportion of respondents declaring a high frequency of lucid dreams was also similar between separated and widowed: 42.06% and 41.18%, respectively. During the pandemic, declaring a high frequency of lucid dreams became more frequent in all categories of marital status, although the increment was more subtle (1.11% increase) among married respondents, it became more noticeable (11.76% enhancement) among widowed peers. For single and separated participants, the increment in the proportion of high lucid dreamers during the pandemic was intermediate (approximately 4%). However, it is worth noting that the sample is considerably unbalanced regarding the abundance of respondents in each category of marital status, raising suspicion about the precision of some estimates.

The frequency of lucid dreams, in terms of proportion of high dreamers, recalled before the pandemic seemed to be proportional to the perception of a financial crisis caused by covid-19 (Figure S2-F). Or in a timely reasonable order, the complaints of economic loss were bigger among participants reporting a high frequency of lucid dreams as compared to low lucid dreamers. However, such a monotonic relation seemed to be abolished during the pandemic. In this context, the proportion of high lucid dreamers was greatest among respondents declaring a considerable economic loss because of covid-19 (61.54%) and lowest on the subset of participants reporting no crisis at all and a lot of financial damage (both with 48.98% of high lucid dreamers). In turn, the abundance of participants reporting a high frequency of lucid dreams was intermediate on categories reflecting a little and a lot of economic loss due to pandemic (53.40% and 56.82%, respectively). Once again, the sample becomes considerably unbalanced when conditioned to the perception of the financial crisis, so that the subsets defined by each category provide heterogeneous levels of confidence.

As illustrated in Figure S3, the (heterogeneous / polychoric) correlation among pairs of demographic variables was found to be, in general terms, negligible. The exceptions were related to the relation among age, marital status, and education, hence precluding the inclusion of all such variables in the same statistical model. As expected, age was positively correlated with education (corr = 0.46), but inversely correlated with marital status (corr = -0.43). In addition, a correlation between education and marital status was also negative (corr = -0.39). There were also some faint correlations from education regarding the number of residents and perception of economic loss due to pandemic (corr = -0.19 and corr = -0.17, respectively). In addition, COVID-19 diagnosis tended to reduce as quarantine enhanced (corr = -0.14) and it was prone to increase as more infected people were known (corr = 0.17). Although such relations endorsed by small coefficients are more likely to be spurious. Importantly, no demographic predictor presented an appraisable correlation with the (binary) frequency of lucid dreams (Figure S3, last three rows/columns). In this sense, it becomes unlikely that these variables could considerably modulate the effect of the pandemic on the frequency of lucid dreams.


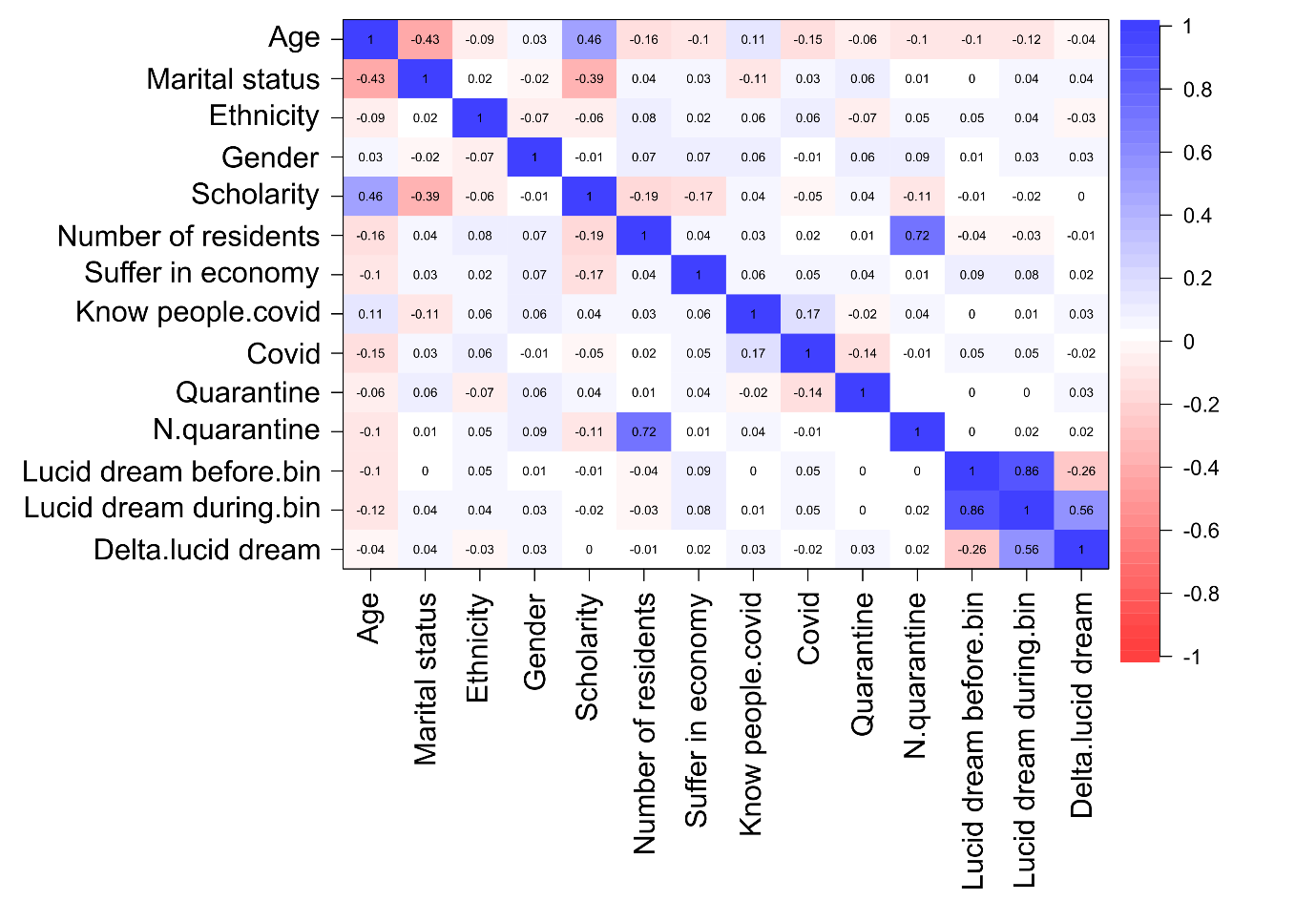


Figure S3 - Polychoric correlation matrix for the demographic predictors along with the frequency of lucid dreams.

In order to evaluate if the data matched the assumptions from the statistical model introduced in Table 1, we proceeded with the analysis of the residues originated from this model. Accordingly, residues were compatible with expected distribution and lacked evidence for overdispersion (Figure S4-A). In addition, residues looked independent and in compliance with homoscedasticity (Figure S4-B). As a result, the model was validated, attesting the inferences derived from it.


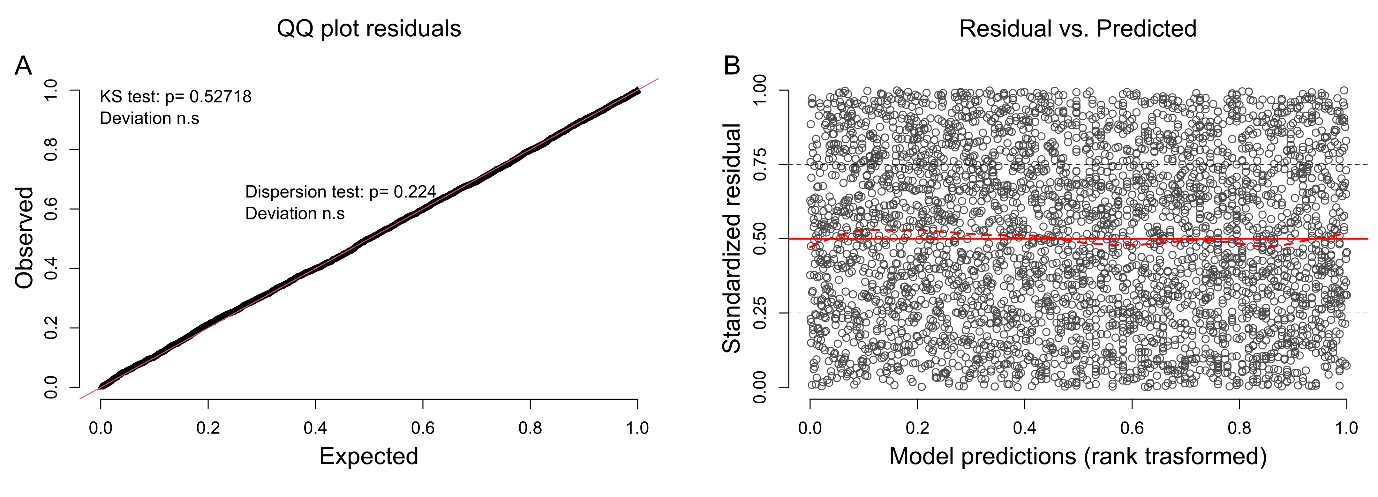


Figure S4 - Residual analysis for the mixed logistic regression model for the frequency of lucid dreams in terms of the pandemic, respondent’s age and the number of residents.

*Factors influencing an increase in lucid dreams frequency*

At first, a descriptive analysis suggests that apnea, insomnia, acting while sleeping, snoring, anxiety, depression or other disorders are likely unimportant to explain the enhancement of lucid dreams frequency during pandemic (Figure S5-A and Figure S5-B). Likewise, the proportion of respondents who reported an increase on the frequency of lucid dreams looked similar across the levels of distress caused by anhedonia or worry (Figure S5-C) and such a proportion seemed to be insensitive to life and health quality (Figure S5-D).

In addition, several issues affecting sleep seem not to elicit, across their levels, a considerable variability regarding the proportion of participants declaring an increase in lucid dreams frequency (Figure S5-E). This is the case for the difficulty in starting and keeping sleeping, waking up too early, feeling asleep or with fatigue, the likelihood of taking a nap and leg discomfort. On the other hand, the frequency of singing while sleeping might be related to an increment in lucid dreams frequency. Sleep singing was quite rare for most respondents, turning the sample considerably unbalanced regarding the levels of this variable, thus eroding much of the confidence on this speculative scrutiny. Nevertheless, the proportion of respondents declaring an increase in the lucid dreams frequency seemed to enhance according to the frequency of sleep singing during the pandemic, from 20.57% among participants who used to sing at night less than monthly up to 62.50% among those reporting to sing three to five times a week. Moreover, there may be a subtle relation between sleep quality (particularly during the pandemic) and the increase in lucid dreams frequency. The proportion of participants declaring that the frequency of lucid dreams increased during the pandemic seemed to be relatively stable across the lowest categories for sleep quality, ranging from 25.00% to 31.65%. However, for those subjects reporting a relatively good or good sleep quality, 18.28% and 11.67% of them, respectively, informed an increment in the frequency of lucid dreams during pandemic.

At last, an exploratory evaluation of the data suggests that the frequency of remembering dreams and nightmares may be related to the recurrence of lucid dreams (Figure S5-F). Especially during the pandemic, the proportion of respondents who declared an increment in lucid dreams frequency gradually increased as remembering the dreams became more usual. Among participants reporting remembering less than one dream a month, 9.35% of them experienced an increase in lucid dreams frequency during the pandemic. Such a proportion mounted up, achieving 32.45% of those who remembered three to five dreams a week, then reducing to 26.50% for participants remembering dreams (almost) daily. The same pattern was observed when the frequency of remembering nightmares was taken into account. For the respondents who remembered less than one nightmare a month, 10.81% of them experienced an increment in lucid dreams frequency. This proportion progressively increased as remembering nightmares became more usual, achieving a maximum of 41.62% of the participants, whose nightmares were remembered from three to five times a week. Among subjects declaring (almost) daily frequency of nightmares, the proportion that experienced an increase in the frequency of lucid dreams was slightly lower, 37.78%.


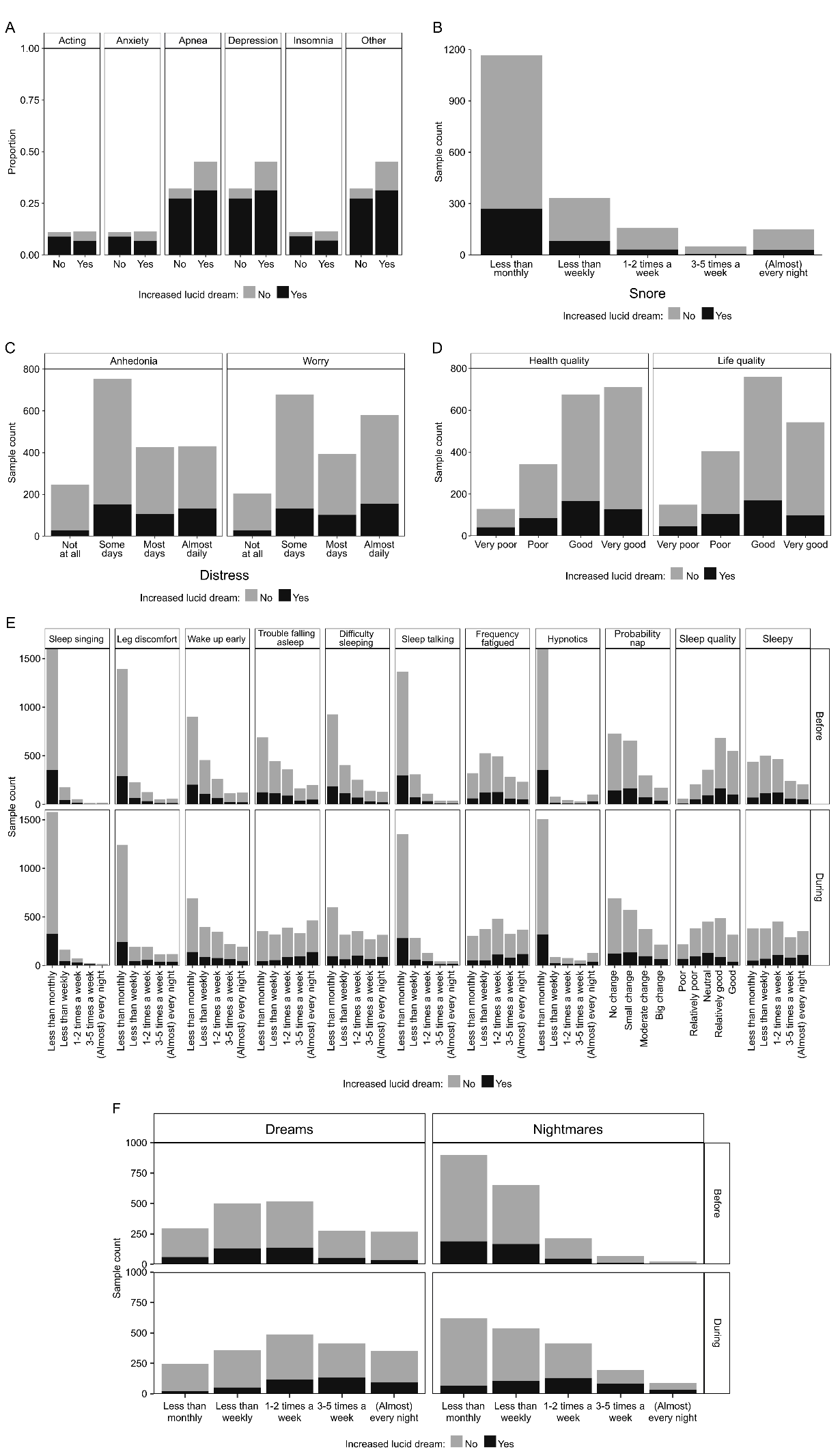


Figure S5 - Descriptive analysis of the sample distribution of increased lucid dreams during the pandemic as conditioned to the levels of events or symptoms related to sleep.

In brief, given the descriptive analysis introduced above, we expect that the frequency of remembering nightmares and dreams are the main factors (from the dataset) influencing the probability of an increment in the recurrence of lucid dreams during the pandemic. This expectation is reinforced by a hierarchical clustering of variables, showing that (along with respondent’s age and number of residents) the frequency of remembering nightmares and dreams have the shortest distances to the increase in lucid dreams frequency (Figure S6). In other words, as compared to the binary scale to measure the increase in lucid dreams during the pandemic, the frequency of remembering dreams and nightmares present the highest similarities, hence carrying more similar information and higher capacity of prediction. In addition, the frequency of sleep singing and the quality of sleep may have some influence on the reported increment of lucid dreams frequency.


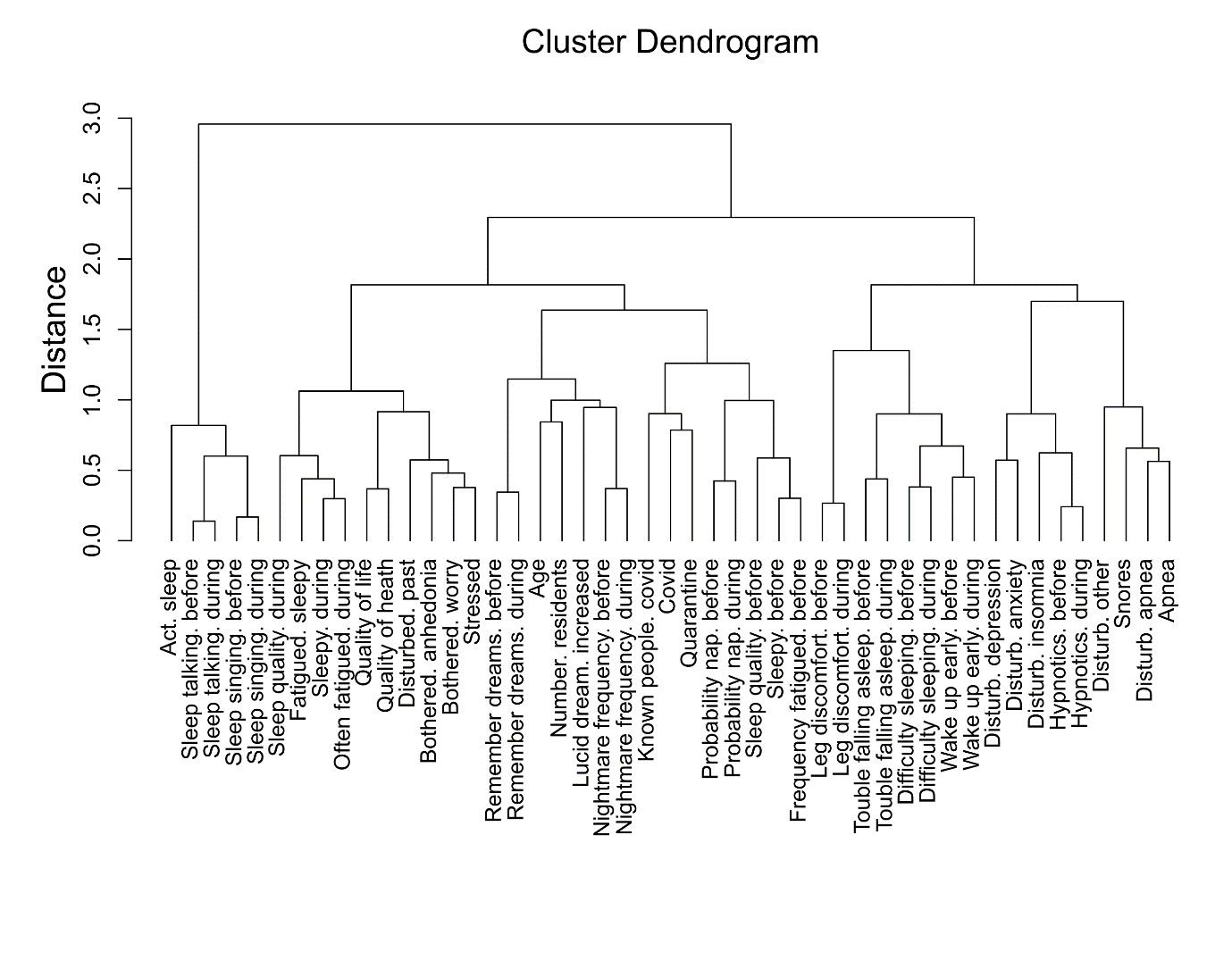


Figure S6 - Dendrogram for hierarchical clustering of variables according to their distance.

In sequence, Figure S7 presents the diagnostic measures for the logistic regression model accounting for the probability of increased lucid dreams frequency according to changes in the frequency of remembering dreams and nightmares, sleep singing and sleep quality during the pandemic. At first, we notice that the leverage of any point is considerably low (Figure S7-A), implying that no observation had an overwhelming weight on the estimation of its own value. In other words, all points counted on a lot of information (besides itself) to estimate its value. Despite the respective low values for Cook’s distance, the observations with relatively higher influence on the estimation of the parameters from the model were highlighted according to their index in the database (Figure S7-B). These observations were later used to substantiate a confirmatory analysis. At last, we identified several conditional outliers (given the model; Figure S7-C), however, the proportion of observations with poor prediction by the model was as low as 2.69%, which could be easily explained by the chance.


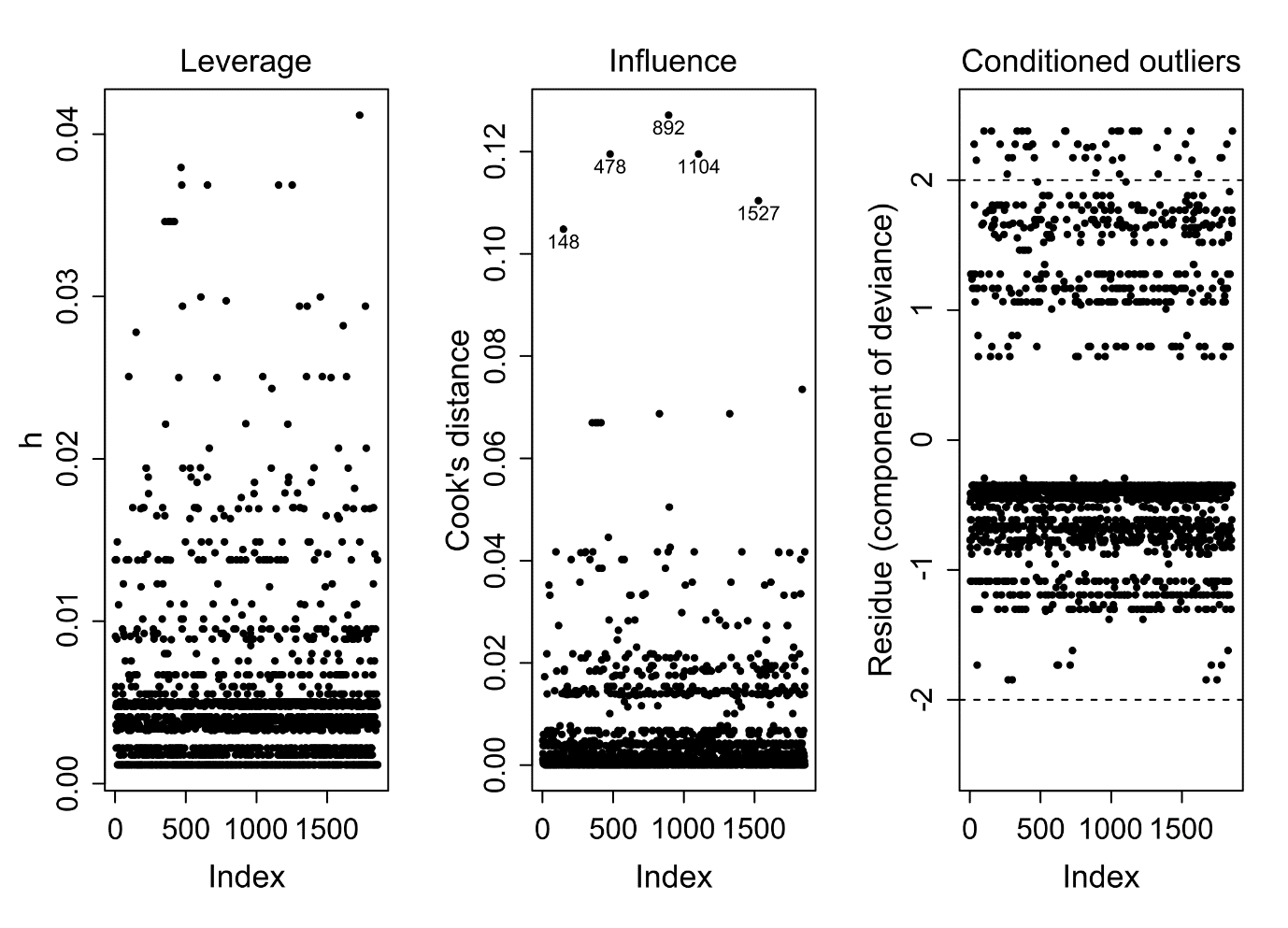


Figure S7 - Diagnostic measures for the logistic regression model for the probability of increased frequency of lucid dreams during the pandemic in terms of changes in the frequency of remembering dreams, nightmares, sleep singing and sleep quality during the pandemic. In A, the higher the value for h, the lower is the abundance of information from other points to predict the response for the corresponding observation. In B, the influence of each observation on the estimation of the model's parameters is exhibited in terms of Cook’s distance. In C, the residues are plotted according to their indices, indicating how far the fitted values are from the observed response for the corresponding observation. Thus, large absolute values for residues indicate poor fitting for respective units.

For confirmatory analysis, the observations found to have higher influence on the estimation of the model’s parameters (in terms of Cook’s distance) were individually and sequentially removed from the dataset, in order to evaluate how much its absence changed the magnitude and inference of the estimated parameters. In general, specific deletion of individual prominent observations had minor influence on the magnitude of the estimates of the parameters, changing their values by less than 5% (Table S1). The exception was the coefficient addressing the effect of a reduction in the frequency of sleep singing during the pandemic, whose value decreased about 30% after the deletion of each of the influential observations. However, none of these units changed the inference for the parameters when dropped. In other words, the effects lacking evidence remained devoid of statistical significance, whereas none of the relevant effects had their statistical significance dependent on individual observations. Thus, the conclusion derived from the model had negligible bias from unusual observations.

|  | | | | | | | | | | | | |
| --- | --- | --- | --- | --- | --- | --- | --- | --- | --- | --- | --- | --- |
| **Coefficients** | **Estimates** | | | | | | **p-value** | | | | | |
|  | **original** | **Δ148** | **Δ478** | **Δ892** | **Δ1104** | **Δ1527** | **original** | **-148** | **-478** | **-892** | **-1104** | **-1527** |
| **(Intercept)** | -2.256 | -0.005 | 0.000 | 0.000 | 0.000 | -0.005 | 0.000 | 0.000 | 0.000 | 0.000 | 0.000 | 0.000 |
| **Nightmares: reduced** | 0.331 | -0.045 | -0.057 | -0.016 | -0.057 | -0.007 | 0.238 | 0.218 | 0.213 | 0.231 | 0.213 | 0.235 |
| **Nightmares: increased** | 1.188 | -0.010 | -0.009 | -0.007 | -0.009 | 0.005 | 0.000 | 0.000 | 0.000 | 0.000 | 0.000 | 0.000 |
| **Dreams: reduced** | 0.305 | -0.016 | -0.012 | -0.055 | -0.012 | -0.056 | 0.125 | 0.119 | 0.121 | 0.106 | 0.121 | 0.106 |
| **Dreams: increased** | 1.351 | 0.005 | 0.005 | -0.010 | 0.005 | -0.009 | 0.000 | 0.000 | 0.000 | 0.000 | 0.000 | 0.000 |
| **Singing: reduced** | -0.366 | -0.313 | -0.341 | -0.347 | -0.341 | -0.322 | 0.331 | 0.220 | 0.210 | 0.208 | 0.210 | 0.217 |
| **Singing: increased** | 1.201 | 0.003 | 0.004 | 0.000 | 0.004 | -0.001 | 0.000 | 0.000 | 0.000 | 0.000 | 0.000 | 0.000 |
| **Sleep:**  **poor** | -0.253 | 0.034 | -0.010 | -0.005 | -0.010 | 0.040 | 0.105 | 0.118 | 0.102 | 0.104 | 0.102 | 0.121 |
| **Sleep:**  **good** | -0.506 | 0.025 | -0.017 | -0.022 | -0.017 | 0.021 | 0.002 | 0.002 | 0.001 | 0.001 | 0.001 | 0.002 |

Table S1 - The confirmatory analysis for the logistic regression model predicting the probability of increased lucid dreams frequency during the pandemic as a function of changes in the frequency of remembering dreams and nightmares, sleep singing and sleep quality during the pandemic. Such an analysis investigates the influence of individual observation by removing it from the dataset and measuring the resulting oscillation on the estimation and inference of the model’s parameters. The Estimates represents a proportional deflection in the magnitude of the coefficient specified in each roll after removing the observation indicated in the header of the column. Following the original values (from the full dataset), the raw p-values for each coefficient are presented in the column specifying the observation that was removed.

At last, we found that the residues derived from the logistic regression model closely matched the expected behavior in adherence with the model's assumptions. As illustrated in Figure S8, practically all the residues are confined within the confidence bands of the envelope, reinforcing the validity of the model, hence the conclusion derived from it.


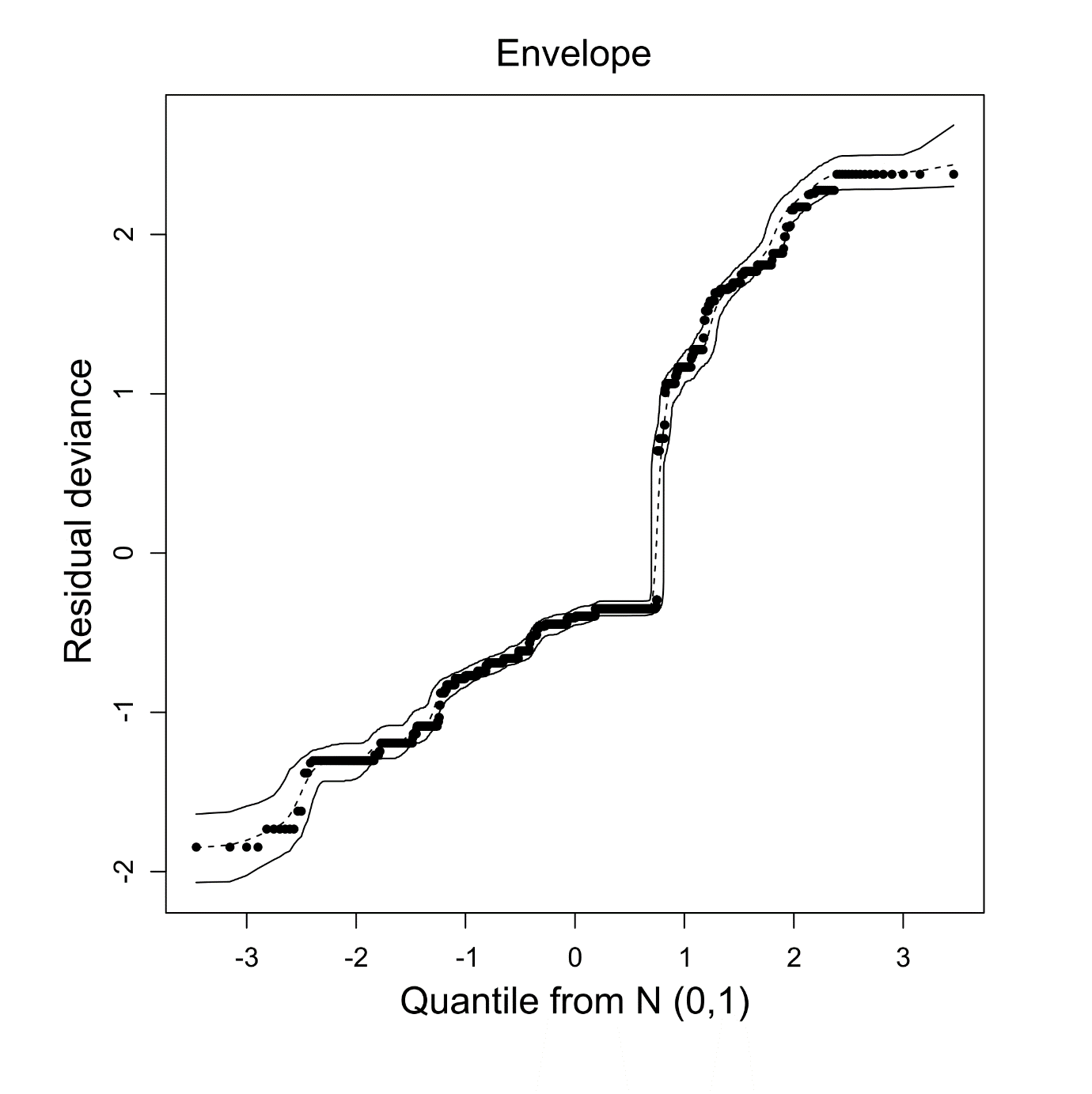


Figure S8 - Residues derived from the logistic regression model in compliance with the expected behavior given the model’s assumptions.
